# A System Bioinformatics Approach Predicts the Molecular Mechanism Underlying the Course of Action of Radix Salviae Reverses GBM Effects

**DOI:** 10.1155/2021/1218969

**Published:** 2021-12-16

**Authors:** Sun Jiaojiao, He Yuping, Li Yajuan, Liu Guangyi, Li Qiuhong, Li Shengbiao, Yu Hong

**Affiliations:** ^1^Department of Histology and Embryology, School of Basic Medical Sciences, Southwest Medical University, Luzhou 646000, Sichuan, China; ^2^Yongchuan Hospital of Chongqing Medical University, Yongchuan 402160, Chongqing, China

## Abstract

**Objective:**

This study used in vitro techniques to investigate the therapeutic effect of Radix Salviae on human glioblastoma and decode its underlying molecular mechanism.

**Methods:**

The active components and targets of the Radix Salviae were identified from the Traditional Chinese Medicine Systems Pharmacology Database (TCMSP). The targets of human glioblastoma were obtained from the GeneCards Database. The Radix Salviae-mediated antiglioblastoma was evaluated by Gene Ontology (GO) analyses and Kyoto Encyclopedia of Genes and Genomes pathway enrichment analyses. Finally, mechanism of action of Radix Salviae against human glioblastoma was deduced by molecular docking and experiments.

**Results:**

We screened 66 active ingredients and 45 targets of the Radix Salviae. The enrichment analysis based on the targets mentioned above suggested a possible role in protein phosphorylation, cell transcription, apoptosis, and inflammatory factor signaling pathways. Further study demonstrated that cryptotanshinone, an essential component of Radix Salviae, played a significant role in killing human glioblastoma cells and protecting the body by inhibiting the AKT, IKB, and STAT3 signaling pathways.

**Conclusions:**

Radix Salviae could inhibit the proliferation and invasion of human glioblastoma by regulating STAT3, Akt, and IKB signaling pathways. Radix Salviae has potential therapeutic value in the future for human glioblastoma.

## 1. Introduction

Human glioblastoma (GBM) is a form of malignant glioma with high mortality and recurrence rates, and the average median survival of patients is less than 18 months [1]. At present, the primary strategy for treating human GBM is surgery combined with radiotherapy or temozolomide (TMZ) chemotherapy [2]. Literature shows that high-dose radiotherapy is likely to damage normal brain tissue [3], and Prolonged TMZ-based chemotherapy causes severe drug resistance and unforeseen side effects [4]. Thus, there is an urgent need to develop alternative treatments targeting GBM.

**Figure 1:**
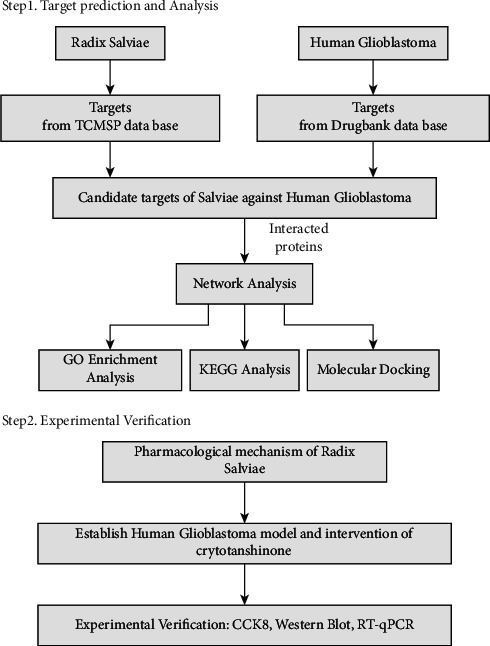
The system-pharmacology framework of Radix Salviae against glioblastoma.

Radix Salviae, a traditional Chinese medicine (TCM), plays a significant role in preventing and treating human GBM [5]. Radix Salviae belongs to the Labiaceae family and has significant clinical value. It has been used for centuries in Asian countries as an antioxidant, anticancer, and anti-inflammatory agent [6]. Cryptotanshinone (CPT) is a crucial active chemical of a natural compound with antitumor activity, which was extracted from the root of Radix Salviae [7]. Although it was widely used in treating cardiovascular and cerebrovascular diseases in the past, Radix Salviae has recently been discovered to have antitumor potential [8]. However, the complex mechanism of action of Radix Salviae against GBM has not yet been fully elucidated. Therefore, the mechanism requires investigation from a network pharmacology perspective. This study used U251 and U87 GBM cell lines to construct a glioblastoma model [9, 10]. We used network pharmacology based on database retrieval, high-throughput combined data analysis, and computer simulation methods to construct a drug-target-disease” network system, which provides a novel strategy for exploring the drug targets for treating the disease [11]. Therefore, our research focused on the glioma target library constructed in the early stage of our laboratory. The targets of human GBM and Radix Salviae were intersected to build a regulatory network of TCMs. In addition, we experimentally verified how Radix Salviae mediates its beneficial effects on GBM. The graphical abstract of this study is shown in (Figure 1).

## 2. Materials and Methods

### 2.1. Active Components and Potential Targets of Radix Salviae

Traditional Chinese Medicine Systems Pharmacology Database and Analysis Platform (TCMSP) was selected to search for the active components of the drug. TCMSP database (https://tcmspw.com/tcmsp.php) is a unique Chinese herbal medicine system pharmacology platform that captures the relationship between drugs, targets, and diseases. Clinically, oral bioavailability (OB) [12] and drug-likeness (DL) [13] were selected as conditions for screening Chinese medicine ingredients. In this study, we first copied the active ingredients of the Chinese medicine (Radix Salviae) from the database to the corresponding file. The drug selection criterion was set to OB ≥ 30% and DL ≥ 0. 18 (Supplementary Table 1) [14]. All target information related to the active ingredient was obtained from the TCMSP database [15].

### 2.2. Potential Targets Screening for GBM

Disease-connected targets were submitted by the GeneCards Database (https://www.genecards.org/), which integrated numerous literature information, covered the analysis date of genes in multiple databases, and included all information on the related genes, making an optimal solution for searching human genetic data. We fetched the targets of GBM from this database.

### 2.3. Network Model Construction

Drugs and disease crossover genes were screened. In the light of the preceding procedure, two sets of input files were prepared: drug-related targets and disease genes. The common genes were leached with R software (https://www.r-project.org/) using the Venn diagram package. The string 11. 0 (https://www.string-db.org/) database was used to obtain proteins related to the target function and eliminate repetitive interactions. Finally, a protein-protein interaction network (PPIs) was constructed using Cytoscape 3. 7. 0.

### 2.4. Enrichment Analysis

Gene Ontology (GO) functional enrichment and Kyoto Encyclopedia of Genes Genomes (KEGG) pathway enrichment was performed using the GO database (https://www.genome.jp/kegg/), and the final results were the macroscopic ones after integration. The common targets of the ingredients and the diseases of the Go enrichment analysis and KEGG path enrichment analysis were obtained using the DAVID database (https://david.ncifcrf.gov/). A histogram was drawn based on the analysis results.

### 2.5. Molecular Docking

Molecular docking is a theoretical simulation method that studies the interaction between principal targets and corresponding components with binding mode and affinity prediction. It mainly includes five significant steps: (1) the compound that is to be docked and critical target need to be selected; (2) the secondary structure of the compound in Chem BioDraw 2D software should be drawn and copied to the Chem BioDraw 3D software to convert it into a three-dimensional structure automatically; (3) the appropriate docking protein and ligand from the Uniport website (https://www.uniprot.org/) should be downloaded [16]; (4) the Pymol software is then used to split the protein and ligand and remove water molecules, phosphorylate, and inactive ligands; and (5) using Auto Dock Tools software to convert compounds, ligands, and critical targets into pdbqt format, Autogrid is used to generate receptor networks, search for active pockets, and run Vina to obtain the affinity list.

### 2.6. Regents

CPT was purchased from Selleckchem company (Houston, TX, USA). Anti-STAT3, anti-p-STAT3 (705), anti-AKT, anti-p-AKT (473), anti-IKB, and anti-p-IKB (32) were from Cell Signaling Technologies (Danvers, MA, USA). Anti-GAPDH was from KangChen company (Shanghai, China).

### 2.7. Cell

Human U87 and U251 glioma cell lines were purchased from the Shanghai Institute of Cell Biology, Chinese Academy of Sciences (Shanghai, China). All the cells were cultured in Dulbecco modified Eagle medium (Gibco, Carlsbad, CA, USA) supplemented with 1% streptomycin/puromycin (HyClone, USA) and 10% fetal bovine serum (Gibco, USA). All cells were maintained at 37°C and 5% CO_2_ in a humidified atmosphere and used for experiments in the midlog phase.

### 2.8. Cell Viability Assay

U251 (5 × 10^3^ cells/well) and U87 (5 × 10^3^ cells/well) GBM cells were seeded onto 96-well, cultured for 48 h after treating with target compounds at various concentrations of CPT (0, 1.25, 2.5, 5, 10, 20 *μ*mol/L). Cellular viability was assayed using a Cell Counting Kit-8 assay (Bimake, Houston, TX, USA). The results were expressed as the absorbance value at 450 nm, which was read using a reader (BioTek Instruments, Winooski, VT, USA).

### 2.9. Western Blotting Analysis

Cells were collected and centrifuged at 200 ×g at 4°C for 5 min to obtain sedimentation. After counting the cells, add protein lysate in equal proportions according to the number of cells to prepare protein samples. Equal amounts of proteins were electrophoresed on 15% sodium dodecyl sulfate polyacrylamide gels based on the molecular weight of the target protein and then transferred to PVDF membranes (Millipore, Billerica, MA, USA). The membranes were blocked with 10% skim milk in Phosphate Buffered Saline-T (0. 5% Tween-20) (PBST) for 1 h at 27°C and then incubated overnight at 4°C with primary antibodies. On the following day, the membranes were washed and further incubated for 1 h in the presence of horseradish peroxidase- (HRP-) conjugated goat anti-rabbit or anti-mouse secondary antibodies at room temperature. Immune reactive protein bands were visualized using UltraSignal chemiluminescence substrate (4A Biotech, Beijing, China), and images were using the MINI Chemi™ 610 chemiluminescent imaging system (Sage, Beijing, China).

### 2.10. RT-qPCR

Total RNAs in U87 and U251 cells were extracted using Trizol Reagent (Solarbio, Shanghai, China), and 800 ng of total RNA was reverse-transcribed to cDNA using a ReverTra Ace qPCR RT Master Mix Kit (TOYOBO, Japan) according to protocol. Real-time PCR was performed with a SYBR1 Green Real-time PCR Master Jit (TOYOBO). CT data were normalized to GAPDH and calculated through a 2^(−△△Ct)^ equation. Primer sequences were listed as follows: IKB; forward primer: ACT ATG CTG AGG TTG GTG TCA TTG G, reverse primer: GGC ACG CTG TTC CAG AGA TTC C; STAT3; forward primer: TCG GCT AGA AAA CTG GAT AAC G, reverse primer: TGC AAC TCC TCC AGT TTC TTA A; AKT; forward primer: GAG GAT CTT CAT GGC GTA GTA G; reverse primer: TGA CCA TGA ACG AGT TTG AGT A; GAPDH; forward primer: ACA ACT TTG GTA TCG TGG AAG G, forward primer: GCC ATC ACG CCA CAG TTT C.

### 2.11. Statistical Analyses

The software of GraphPad Prism 8.0 was applied to statistical analysis. All experiences were performed with triplicate samples and repeated at least three times. Measurement data are expressed as the means ± standard deviation (SD). One-way ANOVA compares differences between groups, followed by an honest significant difference test. *P* values < 0. 05 were considered statistically significant.

## 3. Results

### 3.1. Identification of the Potential Targets and Active Ingredients of Radix Salviae

**Figure 2:**
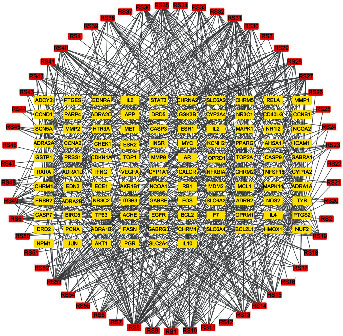
The active component-target network of Radix Salviae. The yellow nodes represented the target genes of Radix Salviae, and the red nodes represented the active ingredient.

TCMSP is an efficient system pharmacology platform that integrates medicinal chemistry, drug similarity, drug targets, related diseases, and interaction networks [17]. According to the screening criteria, 65 active ingredients were identified, and pertinent targets were found in the TCMSP database. The composition-target network diagram of Radix Salviae was made by Cytoscape 3. 7. 0 software (Figure 2). In Figure 2, there are 45 nodes and 175 edges. The average node degrees are 7.78. The Red Rectangle in the outer circle represented active ingredients, and the yellow nodes represented the target gene of Radix Salviae. Because of the large number of ingredients, we numbered MOLID to help facilitate visualization. The specific corresponding details are shown in Supplementary Table 2.

### 3.2. Targets Are Interrelated to Radix Salviae Treatment of Human GBM

**Figure 3:**
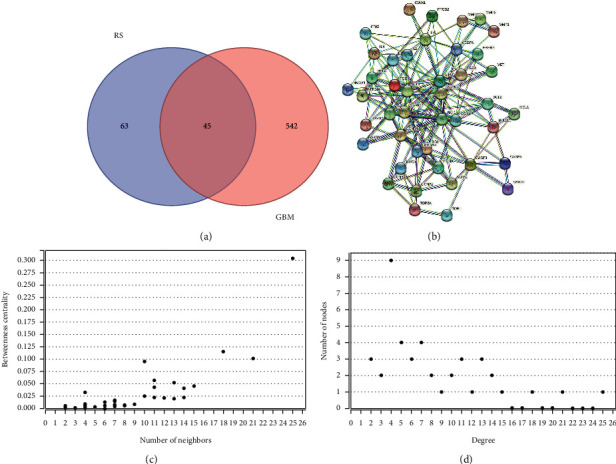
(a) Common Gene Targets of Radix Salviae and human glioblastoma. (b) Interaction network of targets for Radix Salviae against human glioblastoma. (c) Centrality. (d) Distribution.

GeneCards (https://www.genecards.org/) is a gene function query database that summarizes many network databases. The disease targets (690 total) were obtained from the GenenCards database after reduplication. We obtained the intersection targets of active ingredient and disease through R software. The Venn diagram showed 45 essential target genes for Radix Salviae involved in mediating its antihuman GBM effects (Figure 3(a)). The 45 targets were fed into the STRING database (https://string-db.org/) to draw protein-protein interaction (PPI) Networks (Figure 3(b)), and the results were saved as a tsv” file. Then, the file was processed in Cytoscape (version 3. 7. 0) to construct the fundamental target diagram (Figure 3(b)). From this analysis, we found 43 nodes and 175 edges. The average node degree was 8.14, and the average clustering coefficient was 0.59. The specific analysis results are shown in Figures 3(c) and 3(d). The degree of a node corresponded to the number of edges between nodes in the network. The more nodes, the higher the level, and the more pronounced the role of this target in the disease. Betweenness Centrality (BC) represented the probability of the signal going through the node. The higher the BC value, the more the number of neighbors, and hence the more prominent the node [18].

### 3.3. Biological Function and Pathway Enrichment Analyses

#### 3.3.1. GO Analyses

**Figure 4:**
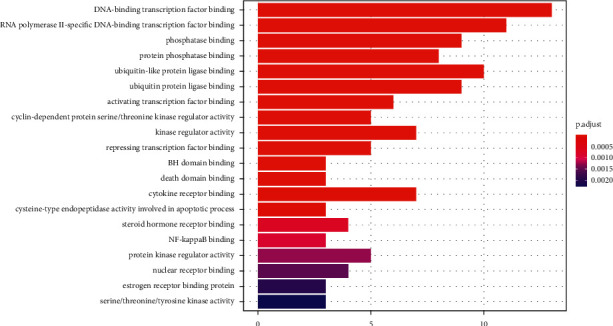
GO analysis for the key targets of Radix Salviae against glioblastoma. *Note*. Molecular function (*y*-axis), gene number (*x*-axis), and *P* value (color).

As shown in Figure 4, the GO analysis revealed that the biological process of predicting key targets was mainly about DNA-binding transcription factor binding, RNA polymerase II-specific DNA-binding transcription factor binding, ubiquitin-like protein ligase binding, phosphatase binding, ligase binding, protein phosphatase binding, kinase regulator activity, cytokine receptor binding, activating transcription factor binding, and so on. In short, these biological processes were mainly of inflammatory factors, DNA transcription, and apoptosis.

#### 3.3.2. Pathways of the RS-HG Target Network

**Figure 5:**
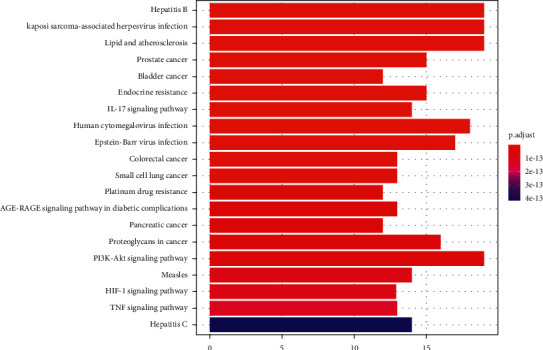
History of KEGG pathway analysis. *Note*. Pathway (*y*-axis), gene number (*x*-axis), and *P* value (color).

**Figure 6:**
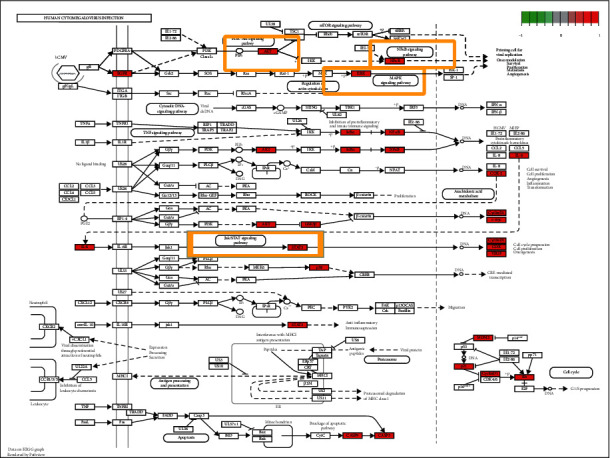
Human cytomegalovirus infection pathway, the important target genes were mainly distributed in the P13K-AKT signaling pathway, the NF-kB signaling pathway, the MAPK signaling pathway, the JAK/STAT signaling pathway. Arrows represented activation impact, T-arrows represented inhibition impact, and segment showed activation or inhibition impact.

The molecular signaling pathways of hub targets in Radix Salviae against human GBM were intently interrelated with hepatitis B, Kaposi sarcoma-associated herpesvirus infection, lipid and atherosclerosis, PI3K-Akt signaling pathway, human cytomegalovirus (HCMV) infection, Epstein–Barr virus infection, proteoglycans in cancer, prostate cancer, endocrine resistance, IL-17 signaling pathway, measles, hepatitis c, colorectal cancer, small cell lung cancer, AGE-RAGE signaling pathway in diabetic complications, HIF-1 signaling pathway, TNF signaling pathway, and so on (Figure 5). HCMV infection was considered to be the leading cause of human GBM. Our KEGG analysis showed that target genes are mainly distributed in the Human cytomegalovirus infection pathway (Figure 6). Follow-up focused on P13K-AKT signaling pathway, NF-*κ*B signaling pathway, and JAK/STAT signaling pathway for performing molecular docking and experimental verification.

#### 3.3.3. Docking the Principal Molecules with the Main Target Proteins

**Figure 7:**
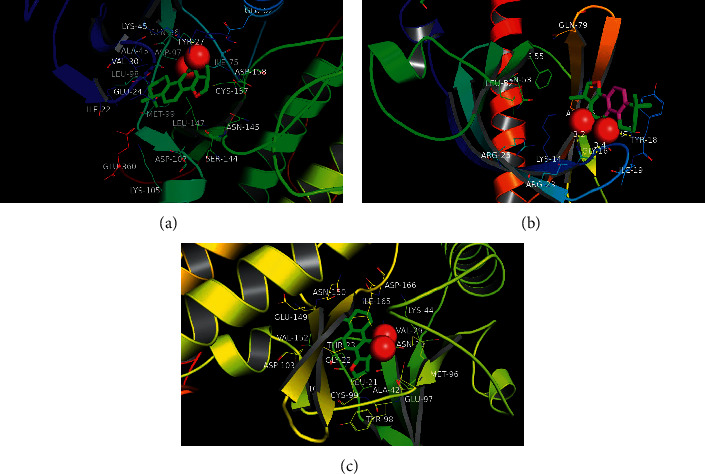
Docking between major molecules and key proteins. (a) Molecular docking model of cryptotanshinone and the Signal Transducer and Activator of Transcription 3. (b) Molecular docking model of cryptotanshinone and protein kinase B. (c) Molecular docking model of cryptotanshinone and I-kappa-B-alpha.

CPT with the highest ingredient score was docked with the Signal Transducer and Activator of Transcription 3 (STAT3) in the network whose binging energy was −8.8 kcal/mol. CPT was also docked with protein kinase B (AKT), and binging energy was −11 kcal/mol. The docking binding energy of CPT and I-kappa-B (IKB) was −10. 5 kcal/mol (Figure 7). The more negative the affinity value, the more reasonable the conformation, suggesting that CPT had the most stable docking with protein kinase B (AKT). Our molecular docking outcome showed a great affinity with the interactions between small molecules and proteins (Table 1).

#### 3.3.4. Antihuman GBM of Cryptotanshinone

**Figure 8:**
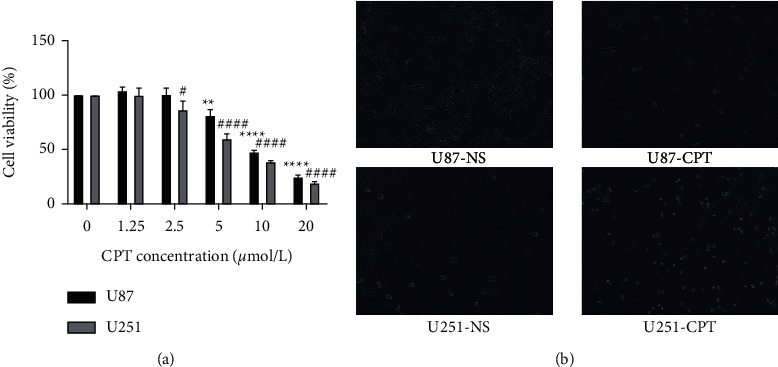
Effect of cryptotanshinone on U87 and U251 cell proliferation (*n* = 3). U87 and U251 cells were processed with cryptotanshinone at 1.25, 2.5, 5, 10, 20 *μ*mol/L for 48 h. The cell viability rate was tested by CCK-8. Cryptotanshinone inhibited the proliferation of both cell lines in a dose-dependent manner. A dose of 10 *μ*mol/L and 20 *μ*mol/L showed the strongest inhibitory effect. (a) CCK-8 results of U87 and U251. (b) Cell state under microscope. Data are given as mean ± SD of individual experiments with five plates in each experiment. ^*∗*^*P* < 0.05 versus 0 *μ*mol/L, ^*∗∗*^*P* < 0.01 versus 0 *μ*mol /L, ^*∗∗∗*^*P* < 0.001 versus 0 *μ*mol /L. ^#^*P* < 0.05 versus 0 *μ*mol /L, ^##^*P* < 0.01 versus 0 *μ*mol /L, ^###^*P* < 0.001 versus 0 *μ*mol/L.

CPT could inhibit the propagation of U87 and U251 cell in a dose-dependent concentration at 48 h. An increased dose led to continuously declining OD values. Compared with 0 *μ*mol/L cryptotanshinone, a high concentration of CPT (20 *μ*mol/L) significantly eased the survival rate of U87 and U251 cells (Figure 8(a)). Significant changes in the cell morphology were also observed at the same time (Figure 8(b)).

#### 3.3.5. Western Blotting Analysis

**Figure 9:**
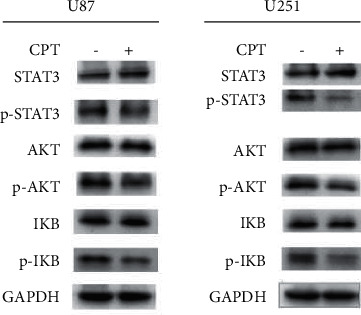
The effects of cryptotanshinone on the expression of p-STAT3, p-AKT, and p-IKB in human glioblastoma. U87 and U251 cells were treated with cryptotanshinone at 20 *μ*mol/L for 48 h. The expression of p-STAT3, p-AKT, and p-IKB was inhibited by the treatment of cryptotanshinone in both cell lines.

We further verified the antitumor mechanism of CPT based on KEGG enrichment analysis and molecular docking results. For example, the therapeutic pathways of CPT on P13K-AKT, NF-*κ*B, and JAK/STAT signaling pathway regulation were checked. For this purpose, we detected the expression level of phosphorylated STAT3, AKT, and IKB (Figure 9). Our results showed CPT exerted antitumor effects by downregulating the phosphorylation of STAT3, AKT, and IKB, which was consistent with the consequences of functional enrichment analysis and molecular docking.

#### 3.3.6. RT-qPCR

**Figure 10:**
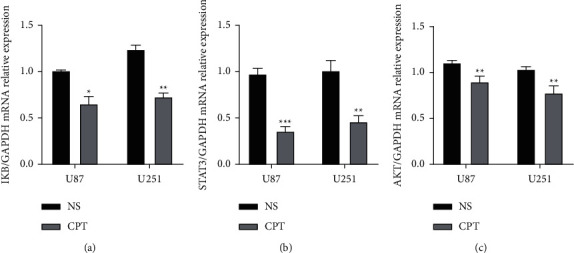
Effects of cryptotanshinone on the relative mRNA expression levels of (a) IKB mRNA, (b) STAT3 mRNA, and (c) AKT mRNA in U87 and U251 cells when compared to control. U87 and U251 cells were treated with cryptotanshinone at 20 *μ*mol/L for 48 h. The values are presented as the mean ± SEM (*n* = 5 in each group). ^*∗*^*P* < 0.05, ^*∗∗*^*P* < 0.01, and ^*∗∗∗*^*P* < 0.001.

The inhibitory effect of CPT on human GBM could be mediated by several pathways, including the IKB, STAT3, AKT. Therefore, we further investigated whether the mRNA expression of U87 and U251 cells was inhibited through quantitative real-time PCR. We found that IKB, STAT3, and AKT expression was decreased significantly upon CPT treatment (Figure 10).

## 4. Discussion

GBM is the deadliest primary malignant tumor among human tumors; it mainly occurs in the central nervous system and is well known for its high recurrence, poor prognosis, and strong lethality. Its clinical symptoms include headache, vomiting, disturbance of consciousness, and speech disturbance [19]. The overall philosophy, syndrome differentiation, and treatment using Chinese medicine have multiple common aspects with the emerging network pharmacology, which complies with the requirements of systematically overcoming complex and miscellaneous diseases. TCM has been previously used to treat GBM as per literature. Berberine inhibits GBM angiogenesis by targeting the VEGFR2/ERK pathway [20]. Shikonin downregulates phosphorylated PI3K/Akt in glioma cell lines U87 and U251 cells [21]. Strychnine induces U251 cell apoptosis and has a killing effect on transplanted tumors [22]. Based on the network pharmacological analysis, this is the first study to explore molecular docking and experimental verification of the potential therapeutic targets of effective components of Radix Salviae on human GBM.

### 4.1. Potential Targets Analysis

Through the target interaction network, we screened out 15 Radix Salviae antihuman GBM key targets (MMP2, MMP1, MET, NF-*κ*BI, AMMP9, PTGS2, IFNG, ICAM1, IL-4, PPARG, HMOX1, IL-6, ERBB2, RELA, and FOS), the condition being node ≥100. Literature shows that the above targets are deemed to be closely related to human GBM. Matrix metalloproteinases 2 (MMP2) promote endothelial cell mitosis and permeability, the degradation of extracellular matrix, and plays a vital role in promoting GBM cell invasion [23]. MMP1 plays a critical role in mobilizing the transfer of human bone marrow-derived mesenchymal stem cells (MSCs) [24]. MET is a high-affinity HGF tyrosine kinase receptor composed of *α* and *β* subunits. The literature shows that MET signaling regulation is the new targeted therapy in GBM treatment [25]. NF-*κ*B activation is an essential driving factor for the malignant phenotype of patients with GBM, which makes the prognosis negative. NFKB1 -94ins/delATTG polymorphism is produced in association with an increased risk of glioma [26]. Previous studies have shown that the high expression of MMP9 in tissues is an independent predictor of survival for patients with WHO grade III glioma tumors. Overexpression of MMP9 can promote the growth of U87 GBM cells and induce a significant increase in their clonogenicity, suggesting that the MMP9 gene may be involved in the occurrence and disease progression of glioma [27]. The author selected 199 adult glioma patients confirmed by histology and 199 cancer-free controls as the objects of this study and analyzed the distribution of PTGS2 genotypes and haplotypes. The results showed that the PTGS2 gene polymorphism might be linked to the susceptibility of glioma in the Chinese population [28]. Interferon-*γ* (IFNG) enhances immune function but promotes T cell failure through PDL1 [29]. In addition to tumor IFNG signal, inhibiting the IFN-I signal in tumor cells might also reduce the expression of drug resistance-related genes. In some cases, knocking out IFNGR alone might lead to a greater antitumor response [30]. GBM cells can infiltrate into healthy brain regions and have dry and aggressive tumor characteristics. Studies have shown that Musashi-1 (MSI1) is a neural stem cell marker that can maintain tumor stem cell status and cell differentiation. Moreover, it also plays an essential role in tumorigenesis. The MSI1/ICAM1 pathway plays a vital role in tumor resistance, including increased tumor invasion. MSI1/ICAM1 may be the target of GBM treatment [31]. The IL-4/IL-13 receptor-mediated STAT3 signaling pathway may be involved in the GBM pathogenesis by regulating the expression of the antiapoptotic protein Bcl-2 family [32]. Moreover, PPARG is associated with increased cancer risk, even in esophageal cancer, glioblastoma, and epithelial tumor subgroups [33]. However, for some practical reasons, detailed environmental data is not combined in the literature. The molecular mechanism of PPARG polymorphism and the occurrence and development of cancer has not been elucidated. Heme oxygenase 1, HMOX1, is part of the most upregulated genes, and its induction depends on ROS. Deletion of HMOX1 enhances Chaex-mediated TRAIL sensitization, proving that HMOX1 is a proapoptotic factor [34]. Interleukin- (IL-) 6 is an important cytokine that activates several cancer-promoting signaling pathways in glioblastoma [35]. The authors found that in 41% of native GBM samples and most of the GBM cell lines investigated, ErbB2 protein expression was elevated, thereby inducing endogenous antitumor immunity and having long-term protection against rechallenge of distant tumors [36]. MATN1-AS1 inhibits the proliferation and invasion of glioblastoma cells through RELA regulation [37]. c-Fos inhibits phospholipid synthesis and activation and interferes with the proliferation of glioblastoma cells. Targeting the N-terminal part of c-Fos or shorter derivatives may provide a new therapeutic strategy for treating GBM [38]. Enrichment analyses showed that some critical targets could control multiple pathways. The three pathways, P13K-AKT, NF-*κ*B, MAPK, and JAK/STAT signal pathway, came out to be significant.

### 4.2. Pathway Analysis

HCMV plays a significant role in the proliferation and metastasis of GBM [39]; the article emphasizes that immunomodulatory properties have a close association with the GBM stage. Activation of HCMV promotes the virus from the peripheral blood into essential tissues, inhibits the local immune response, assists GBM tumors in escaping immune surveillance, and leads to a poor prognosis of the disease [40]. Therefore, this study selected this pathway as a vital follow-up research object. Depending on the outcome of the KEGG analysis, we reselected some critical downstream pathways (STAT3, AKT, IKB) according to the literature for molecular docking and cell experiments for verification. The development and progression of GBM tumors are heavily dependent on the activation of STAT3 to promote cell proliferation, and STAT3 phosphorylation is closely related to the GSC phenotype and immune escape by regulating the tumor microenvironment, thereby promoting tumor recurrence and resistance to standard treatments [41]. It has been reported that high levels of phosphocreatine AKT (p-AKT) are associated [42]. Dominant mutations in genes encoding AKT family members have not been observed in human tumors, so the activation of AKT seems to be the result of changes in its upstream molecules [43]. Blocking the AKT signaling pathway can lead to tumor cell apoptosis and growth inhibition. The observed dependence of certain tumors on Akt signaling has a wide-ranging impact on tumor survival and growth [44]. Baicalin can significantly inhibit the change of p-IKB*α*/IKB*α* content but has no significant effect on p-IKK*β*/IKK*β* [45].

### 4.3. Experimental Analysis

U251 and U87 cell lines are frequently used experimental models of glioma, which promote cell proliferation by regulating nicotinamide nucleotide metabolism, RNA splicing, glycolysis, and purine metabolism [46]. Cell proliferation is driven by a series of proteins called cycling, a key component of the cell cycle machinery. They operate in the nucleus, bind and activate cyclin-dependent kinases (CDKs), and provide substrate specificity [47]. CPT significantly inhibits glioma cells' proliferation, spherical growth, colony formation, and stem cell characteristics by inducing apoptosis and cell cycle arrest [7]. Moreover, CPT has been shown to have antitumor activity in various malignant tumors, including breast cancer, liver cancer, prostate cancer, and ovarian cancer [48]. According to literature, CPT blocks the phosphorylation and homodimerization of STAT3 and exerts antitumor effects *in vivo* and *in vitro* by targeting the STAT3/SIRT3/HIF-1*α* signaling axis [49]. In this study, CPT was used to treat human GBM cell lines U87 and U251. CCK8 results showed that CPT was nontoxic for normal cells within an effective dose. However, the most potent inhibitory effect on tumor proliferation appeared in the concentration of 10 and 20 *μ*mol/L. The STAT3 signaling pathway plays a vital role in tumor growth. At the protein level, we explored whether CPT affected the constructional activity of STAT3 in U87 and U251 cells. The phosphorylation of STAT3 was significantly inhibited by CPT (20 *μ*mol/L), but the total protein of STAT3 remained unchanged by CPT. We continue to detect several upstream kinases, particularly those involved in cell proliferation, including IKB and AKT. The results showed that phosphorylation levels were downregulated between the two cell lines. Moreover, the mRNA expression levels of IKB, STAT3, and AKT treated by CPT were detected by quantitative real-time RT- PCR. The results showed that the expression levels of the three genes were downregulated. In summary, CPT has a definite effect on antiglioblastoma.

### 4.4. Value of Research

Identifying bioactive compounds from TCM is an essential method for developing new drug research. At present, several experiments are being conducted to identify active compounds with clinical therapeutic value. However, the process is expensive, lacks efficiency, and requires substantial workforce and material resources. Network pharmacology provides excellent advantages in studying the mode of action of drugs. Crucially, the combination of prediction results and experimental verifications significantly improves the discovery speed of natural compounds. This study predicted the potential molecular mechanism of action of Radix Salviae on human GBM through the computational method. A series of experiments were also devised to verify the predicted results. The results collectively suggested that Radix Salviae exerts antitumor effects through targeting IKB, STAT3, and AKT.

## 5. Conclusions

In the current study, 45 potential targets of Radix Salviae for treating GBM were confirmed by network pharmacology and further validated by molecular docking. In addition, the molecular mechanisms utilized by the conventional GBM therapies were further demonstrated by *in vitro* experiments. Our enrichment analysis showed features of synergistic interaction of multiple targets and suggested that targeting the AKT, STAT3, and IKB pathways could alleviate the symptoms of GBM through protein phosphorylation, apoptosis, and inflammatory factors. Our study provides a better theoretical approach to explore the treatment of human GBM. One drawback of this study remains that some experiments were carried out *in vitro*, and the experimental verification still needs to be done *in vivo*.

## Figures and Tables

**Table 1:** Docking of molecules with the target protein.

Target	PDB ID	Ligand	Three dimensional coordinates of the active site	Molecule	Affinity(kcal/mol)
STAT3	5AX3	5ID	*x* = 16. 833; *y* = -6. 516; *z* = -16. 892	Cryptotanshinone	−8. 8
AKT	1LUNQ	4IP	*x* = 53. 954; *y* = 20. 789; *z* = 83. 704	Cryptotanshinone	−11
IKB	4KIK	KSA	*x* = -0. 637; *y* = 1. 085; *z* = 0. 694	Cryptotanshinone	−10. 5

## Data Availability

The data used to support the findings of this study are included within the article and supplementary information files.

## Supplementary Materials

Supplementary Materials Supplementary Table 1: potential active components of Radix Salviae. Supplementary Table 2: molecular information and targets corresponding to the coding ID.
