# Supplementary material for: A System Bioinformatics Approach Predicts the Molecular Mechanism Underlying the Course of Action of Radix Salviae Reverses GBM Effects
Source: Evid Based Complement Alternat Med. 2021 Dec 16;2021:1218969. doi: 10.1155/2021/1218969 (PMC8825271; doi:10.1155/2021/1218969)
Supplement: Supplementary Materials — Supplementary Table 1: potential active components of Radix Salviae. Supplementary Table 2: molecular information and targets corresponding to the coding ID. [file 1218969.f1.zip › 1218969.f1/Supplementary Table 2.docx]

| ID | MolId | Target | ID | MOLD | Target |
| --- | --- | --- | --- | --- | --- |
| RS1 | MOL001601 | PTGS1 | RS29 | MOL007094 | OPRM1 |
| RS1 | MOL001601 | CHRM3 | RS29 | MOL007094 | GABRA1 |
| RS1 | MOL001601 | CHRM1 | RS30 | MOL007098 | PTGS1 |
| RS1 | MOL001601 | SCN5A | RS30 | MOL007098 | CHRM3 |
| RS1 | MOL001601 | CHRM5 | RS30 | MOL007098 | CHRM1 |
| RS1 | MOL001601 | PTGS2 | RS30 | MOL007098 | ESR1 |
| RS1 | MOL001601 | HTR3A | RS30 | MOL007098 | AR |
| RS1 | MOL001601 | CHRM4 | RS30 | MOL007098 | SCN5A |
| RS1 | MOL001601 | RXRA | RS30 | MOL007098 | CHRM5 |
| RS1 | MOL001601 | OPRD1 | RS30 | MOL007098 | PTGS2 |
| RS1 | MOL001601 | ADRA1A | RS30 | MOL007098 | CHRM4 |
| RS1 | MOL001601 | CHRM2 | RS30 | MOL007098 | RXRA |
| RS1 | MOL001601 | ADRA1B | RS30 | MOL007098 | OPRD1 |
| RS1 | MOL001601 | SLC6A3 | RS30 | MOL007098 | ADRA1A |
| RS1 | MOL001601 | ADRB2 | RS30 | MOL007098 | CHRM2 |
| RS1 | MOL001601 | ADRA1D | RS30 | MOL007098 | ADRA1B |
| RS1 | MOL001601 | OPRM1 | RS30 | MOL007098 | ADRB2 |
| RS1 | MOL001601 | GABRA1 | RS30 | MOL007098 | ADRA1D |
| RS1 | MOL001601 | NCOA2 | RS30 | MOL007098 | OPRM1 |
| RS1 | MOL001601 | NCOA1 | RS30 | MOL007098 | GSK3B |
| RS1 | MOL001601 | SLC6A4 | RS30 | MOL007098 | NCOA2 |
| RS2 | MOL001659 | PGR | RS30 | MOL007098 | NCOA1 |
| RS2 | MOL001659 | NR3C2 | RS31 | MOL007100 | NOS2 |
| RS3 | MOL001771 | PGR | RS31 | MOL007100 | PTGS1 |
| RS3 | MOL001771 | NCOA2 | RS31 | MOL007100 | CHRM3 |
| RS4 | MOL001942 | PTGS2 | RS31 | MOL007100 | CHRM1 |
| RS5 | MOL002222 | CHRM3 | RS31 | MOL007100 | ESR1 |
| RS5 | MOL002222 | CHRM1 | RS31 | MOL007100 | AR |
| RS5 | MOL002222 | SCN5A | RS31 | MOL007100 | SCN5A |
| RS5 | MOL002222 | CHRM5 | RS31 | MOL007100 | PPARG |
| RS5 | MOL002222 | PTGS2 | RS31 | MOL007100 | CHRM5 |
| RS5 | MOL002222 | CHRM4 | RS31 | MOL007100 | PTGS2 |
| RS5 | MOL002222 | OPRD1 | RS31 | MOL007100 | HTR3A |
| RS5 | MOL002222 | ACHE | RS31 | MOL007100 | RXRA |
| RS5 | MOL002222 | ADRA1A | RS31 | MOL007100 | ACHE |
| RS5 | MOL002222 | CHRM2 | RS31 | MOL007100 | ADRA1A |
| RS5 | MOL002222 | ADRA1B | RS31 | MOL007100 | ADRA1B |
| RS5 | MOL002222 | ADRB2 | RS31 | MOL007100 | ESR1 |
| RS5 | MOL002222 | ADRA1D | RS31 | MOL007100 | AR |
| RS5 | MOL002222 | DRD2 | RS31 | MOL007100 | SCN5A |
| RS5 | MOL002222 | OPRM1 | RS31 | MOL007100 | PPARG |
| RS6 | MOL002651 | CHRM3 | RS31 | MOL007100 | CHRM5 |
| RS6 | MOL002651 | CHRM1 | RS31 | MOL007100 | PTGS2 |
| RS6 | MOL002651 | ESR1 | RS31 | MOL007100 | HTR3A |
| RS6 | MOL002651 | AR | RS31 | MOL007100 | RXRA |
| RS6 | MOL002651 | SCN5A | RS31 | MOL007100 | ACHE |
| RS6 | MOL002651 | PPARG | RS31 | MOL007100 | ADRA1A |
| RS6 | MOL002651 | CHRM5 | RS31 | MOL007100 | ADRA1B |
| RS6 | MOL002651 | PTGS2 | RS31 | MOL007100 | SLC6A3 |
| RS6 | MOL002651 | CHRM4 | RS31 | MOL007100 | ADRB2 |
| RS6 | MOL002651 | OPRD1 | RS31 | MOL007100 | ADRA1D |
| RS6 | MOL002651 | ACHE | RS31 | MOL007100 | SLC6A4 |
| RS6 | MOL002651 | ADRA1A | RS31 | MOL007100 | OPRM1 |
| RS6 | MOL002651 | ADRB2 | RS31 | MOL007100 | GABRA1 |
| RS6 | MOL002651 | OPRM1 | RS31 | MOL007100 | GSK3B |
| RS6 | MOL002651 | GABRA1 | RS31 | MOL007100 | PRSS1 |
| RS6 | MOL002651 | NCOA1 | RS31 | MOL007100 | CCNA2 |
| RS7 | MOL000569 | PTGS2 | RS32 | MOL007101 | PTGS1 |
| RS7 | MOL000569 | AKR1B1 | RS32 | MOL007101 | SCN5A |
| RS8 | MOL000006 | PTGS1 | RS32 | MOL007101 | PTGS2 |
| RS8 | MOL000006 | AR | RS32 | MOL007101 | HTR3A |
| RS8 | MOL000006 | PTGS2 | RS32 | MOL007101 | RXRA |
| RS8 | MOL000006 | PRSS1 | RS32 | MOL007101 | ADRA1A |
| RS8 | MOL000006 | NCOA2 | RS32 | MOL007101 | ADRA1B |
| RS8 | MOL000006 | RELA | RS32 | MOL007101 | ADRB2 |
| RS8 | MOL000006 | EGFR | RS32 | MOL007101 | GABRA1 |
| RS8 | MOL000006 | AKT1 | RS32 | MOL007101 | NCOA2 |
| RS8 | MOL000006 | VEGFA | RS32 | MOL007101 | NCOA1 |
| RS8 | MOL000006 | CCND1 | RS33 | MOL007105 | PTGS1 |
| RS8 | MOL000006 | BCL2L1 | RS33 | MOL007105 | CHRM3 |
| RS8 | MOL000006 | CDKN1A | RS33 | MOL007105 | CHRM1 |
| RS8 | MOL000006 | CASP9 | RS33 | MOL007105 | ESR1 |
| RS8 | MOL000006 | MMP2 | RS33 | MOL007105 | SCN5A |
| RS8 | MOL000006 | MMP9 | RS33 | MOL007105 | CHRM5 |
| RS8 | MOL000006 | MAPK1 | RS33 | MOL007105 | PTGS2 |
| RS8 | MOL000006 | IL10 | RS33 | MOL007105 | CHRM4 |
| RS8 | MOL000006 | RB1 | RS33 | MOL007105 | RXRA |
| RS8 | MOL000006 | TNFSF15 | RS33 | MOL007105 | OPRD1 |
| RS8 | MOL000006 | JUN | RS33 | MOL007105 | ADRA1A |
| RS8 | MOL000006 | IL6 | RS33 | MOL007105 | CHRM2 |
| RS8 | MOL000006 | CASP3 | RS33 | MOL007105 | ADRA1B |
| RS8 | MOL000006 | TP63 | RS33 | MOL007105 | ADRB2 |
| RS8 | MOL000006 | NFKBIA | RS33 | MOL007105 | ADRA1D |
| RS8 | MOL000006 | TOP1 | RS33 | MOL007105 | SLC6A4 |
| RS8 | MOL000006 | MDM2 | RS33 | MOL007105 | OPRM1 |
| RS8 | MOL000006 | APP | RS33 | MOL007105 | GABRA1 |
| RS8 | MOL000006 | MMP1 | RS34 | MOL007107 | CHRM3 |
| RS8 | MOL000006 | PCNA | RS34 | MOL007107 | CHRM1 |
| RS8 | MOL000006 | ERBB2 | RS34 | MOL007107 | SCN5A |
| RS8 | MOL000006 | PPARG | RS34 | MOL007107 | ACHE |
| RS8 | MOL000006 | HMOX1 | RS34 | MOL007107 | ADRA1A |
| RS8 | MOL000006 | CASP7 | RS34 | MOL007107 | CHRM2 |
| RS8 | MOL000006 | ICAM1 | RS34 | MOL007107 | ADRA1B |
| RS8 | MOL000006 | MCL1 | RS34 | MOL007107 | ADRB2 |
| RS8 | MOL000006 | BIRC5 | RS34 | MOL007107 | ADRA1D |
| RS8 | MOL000006 | IL2 | RS34 | MOL007107 | OPRM1 |
| RS8 | MOL000006 | CCNB1 | RS35 | MOL007108 | NOS2 |
| RS8 | MOL000006 | TYR | RS35 | MOL007108 | PTGS1 |
| RS8 | MOL000006 | IFNG | RS35 | MOL007108 | CHRM3 |
| RS8 | MOL000006 | IL4 | RS35 | MOL007108 | CHRM1 |
| RS8 | MOL000006 | TOP2A | RS35 | MOL007108 | ESR1 |
| RS8 | MOL000006 | GSTP1 | RS35 | MOL007108 | AR |
| RS8 | MOL000006 | SLC2A4 | RS35 | MOL007108 | SCN5A |
| RS8 | MOL000006 | INSR | RS35 | MOL007108 | CHRM5 |
| RS8 | MOL000006 | CD40LG | RS35 | MOL007108 | PTGS2 |
| RS8 | MOL000006 | PTGES | RS35 | MOL007108 | CHRM4 |
| RS8 | MOL000006 | NUF2 | RS35 | MOL007108 | RXRA |
| RS8 | MOL000006 | ADCY2 | RS35 | MOL007108 | OPRD1 |
| RS8 | MOL000006 | MET | RS35 | MOL007108 | ACHE |
| RS9 | MOL007036 | PTGS1 | RS35 | MOL007108 | ADRA1A |
| RS9 | MOL007036 | CHRM3 | RS35 | MOL007108 | CHRM2 |
| RS9 | MOL007036 | CHRM1 | RS35 | MOL007108 | ADRA1B |
| RS9 | MOL007036 | SCN5A | RS35 | MOL007108 | ADRB2 |
| RS9 | MOL007036 | PTGS2 | RS35 | MOL007108 | ADRA1D |
| RS9 | MOL007036 | RXRA | RS35 | MOL007108 | DRD2 |
| RS9 | MOL007036 | ACHE | RS35 | MOL007108 | OPRM1 |
| RS9 | MOL007036 | ADRA1A | RS35 | MOL007108 | GABRA1 |
| RS9 | MOL007036 | ADRA1B | RS35 | MOL007108 | PRSS1 |
| RS9 | MOL007036 | ADRB2 | RS35 | MOL007108 | NCOA2 |
| RS9 | MOL007036 | OPRM1 | RS35 | MOL007108 | NCOA1 |
| RS9 | MOL007036 | NCOA2 | RS36 | MOL007111 | NOS2 |
| RS9 | MOL007036 | NCOA1 | RS36 | MOL007111 | CHRM3 |
| RS10 | MOL007041 | PTGS1 | RS36 | MOL007111 | CHRM1 |
| RS10 | MOL007041 | CHRM3 | RS36 | MOL007111 | ESR1 |
| RS10 | MOL007041 | CHRM1 | RS36 | MOL007111 | AR |
| RS10 | MOL007041 | ESR1 | RS36 | MOL007111 | SCN5A |
| RS10 | MOL007041 | AR | RS36 | MOL007111 | CHRM5 |
| RS10 | MOL007041 | SCN5A | RS36 | MOL007111 | PTGS2 |
| RS10 | MOL007041 | PPARG | RS36 | MOL007111 | RXRA |
| RS10 | MOL007041 | CHRM5 | RS36 | MOL007111 | OPRD1 |
| RS10 | MOL007041 | PTGS2 | RS36 | MOL007111 | ACHE |
| RS10 | MOL007041 | HTR3A | RS36 | MOL007111 | ADRA1A |
| RS10 | MOL007041 | CHRM4 | RS36 | MOL007111 | CHRM2 |
| RS10 | MOL007041 | RXRA | RS36 | MOL007111 | ADRB2 |
| RS10 | MOL007041 | ADRA1A | RS36 | MOL007111 | OPRM1 |
| RS10 | MOL007041 | CHRM2 | RS36 | MOL007111 | ESR2 |
| RS10 | MOL007041 | ADRA1B | RS36 | MOL007111 | GABRA1 |
| RS10 | MOL007041 | SLC6A3 | RS36 | MOL007111 | GSK3B |
| RS10 | MOL007041 | ADRB2 | RS36 | MOL007111 | CHEK1 |
| RS10 | MOL007041 | ADRA1D | RS36 | MOL007111 | CCNA2 |
| RS10 | MOL007041 | SLC6A4 | RS37 | MOL007115 | NCOA2 |
| RS10 | MOL007041 | OPRM1 | RS38 | MOL007119 | PTGS1 |
| RS10 | MOL007041 | GABRA1 | RS38 | MOL007119 | CHRM3 |
| RS10 | MOL007041 | CCNA2 | RS38 | MOL007119 | CHRM1 |
| RS10 | MOL007041 | NCOA2 | RS38 | MOL007119 | ESR1 |
| RS11 | MOL007045 | CHRM1 | RS38 | MOL007119 | AR |
| RS11 | MOL007045 | SCN5A | RS38 | MOL007119 | SCN5A |
| RS11 | MOL007045 | CHRM5 | RS38 | MOL007119 | PTGS2 |
| RS11 | MOL007045 | PTGS2 | RS38 | MOL007119 | RXRA |
| RS11 | MOL007045 | OPRD1 | RS38 | MOL007119 | OPRD1 |
| RS11 | MOL007045 | ACHE | RS38 | MOL007119 | ADRA1A |
| RS11 | MOL007045 | ADRB2 | RS38 | MOL007119 | CHRM2 |
| RS11 | MOL007045 | OPRM1 | RS38 | MOL007119 | ADRA1B |
| RS11 | MOL007045 | PRSS1 | RS38 | MOL007119 | ADRB2 |
| RS11 | MOL007045 | NCOA1 | RS38 | MOL007119 | OPRM1 |
| RS12 | MOL007048 | PTGS2 | RS38 | MOL007119 | NR3C1 |
| RS13 | MOL007049 | PTGS1 | RS38 | MOL007119 | GSK3B |
| RS13 | MOL007049 | CHRM3 | RS38 | MOL007119 | CCNA2 |
| RS13 | MOL007049 | CHRM1 | RS38 | MOL007119 | NCOA2 |
| RS13 | MOL007049 | ESR1 | RS38 | MOL007119 | NCOA1 |
| RS13 | MOL007049 | AR | RS39 | MOL007120 | PTGS2 |
| RS13 | MOL007049 | SCN5A | RS39 | MOL007120 | ACHE |
| RS13 | MOL007049 | PPARG | RS39 | MOL007120 | PGR |
| RS13 | MOL007049 | CHRM5 | RS39 | MOL007120 | NR3C1 |
| RS13 | MOL007049 | PTGS2 | RS39 | MOL007120 | NCOA2 |
| RS13 | MOL007049 | ADRA2A | RS39 | MOL007120 | NCOA1 |
| RS13 | MOL007049 | ADRA2C | RS40 | MOL007121 | ESR1 |
| RS13 | MOL007049 | CHRM4 | RS40 | MOL007121 | ACHE |
| RS13 | MOL007049 | RXRA | RS40 | MOL007122 | PTGS1 |
| RS13 | MOL007049 | OPRD1 | RS40 | MOL007122 | CHRM3 |
| RS13 | MOL007049 | ADRA1A | RS40 | MOL007122 | CHRM1 |
| RS13 | MOL007049 | CHRM2 | RS40 | MOL007122 | ESR1 |
| RS13 | MOL007049 | ADRA1B | RS40 | MOL007122 | AR |
| RS13 | MOL007049 | SLC6A3 | RS40 | MOL007122 | DRD5 |
| RS13 | MOL007049 | ADRB2 | RS40 | MOL007122 | SCN5A |
| RS13 | MOL007049 | ADRA1D | RS40 | MOL007122 | CHRM5 |
| RS13 | MOL007049 | SLC6A4 | RS40 | MOL007122 | PTGS2 |
| RS13 | MOL007049 | DRD2 | RS40 | MOL007122 | ADRA2C |
| RS13 | MOL007049 | OPRM1 | RS40 | MOL007122 | CHRM4 |
| RS13 | MOL007049 | GABRA1 | RS40 | MOL007122 | RXRA |
| RS13 | MOL007049 | NCOA2 | RS40 | MOL007122 | OPRD1 |
| RS13 | MOL007049 | NCOA1 | RS40 | MOL007122 | ADRA1A |
| RS14 | MOL007050 | NOS2 | RS40 | MOL007122 | CHRM2 |
| RS14 | MOL007050 | ESR1 | RS40 | MOL007122 | ADRA1B |
| RS14 | MOL007050 | AR | RS40 | MOL007122 | SLC6A3 |
| RS14 | MOL007050 | PPARG | RS40 | MOL007122 | ADRB2 |
| RS14 | MOL007050 | ESR2 | RS40 | MOL007122 | ADRA1D |
| RS14 | MOL007050 | MAPK14 | RS40 | MOL007122 | OPRM1 |
| RS14 | MOL007050 | GSK3B | RS40 | MOL007122 | NCOA2 |
| RS14 | MOL007050 | CCNA2 | RS41 | MOL007124 | PTGS1 |
| RS14 | MOL007058 | AR | RS41 | MOL007124 | CHRM3 |
| RS14 | MOL007058 | PTGS2 | RS41 | MOL007124 | CHRM1 |
| RS14 | MOL007058 | RXRA | RS41 | MOL007124 | ESR1 |
| RS14 | MOL007058 | NCOA1 | RS41 | MOL007124 | AR |
| RS14 | MOL007059 | CHRM1 | RS41 | MOL007124 | SCN5A |
| RS14 | MOL007059 | PTGS2 | RS41 | MOL007124 | PTGS2 |
| RS14 | MOL007059 | RXRA | RS41 | MOL007124 | CHRM4 |
| RS14 | MOL007059 | OPRD1 | RS41 | MOL007124 | RXRA |
| RS14 | MOL007059 | ACHE | RS41 | MOL007124 | OPRD1 |
| RS14 | MOL007059 | ADRA1A | RS41 | MOL007124 | ADRA1A |
| RS14 | MOL007059 | ADRB2 | RS41 | MOL007124 | CHRM2 |
| RS14 | MOL007059 | OPRM1 | RS41 | MOL007124 | ADRA1B |
| RS14 | MOL007059 | PRSS1 | RS41 | MOL007124 | SLC6A3 |
| RS14 | MOL007059 | NCOA1 | RS41 | MOL007124 | ADRB2 |
| RS15 | MOL007061 | CHRM3 | RS41 | MOL007124 | ADRA1D |
| RS15 | MOL007061 | CHRM1 | RS41 | MOL007124 | SLC6A4 |
| RS15 | MOL007061 | SCN5A | RS41 | MOL007124 | OPRM1 |
| RS15 | MOL007061 | CHRM5 | RS41 | MOL007124 | GABRA1 |
| RS15 | MOL007061 | PTGS2 | RS41 | MOL007124 | GSK3B |
| RS15 | MOL007061 | RXRA | RS41 | MOL007124 | CCNA2 |
| RS15 | MOL007061 | OPRD1 | RS42 | MOL007125 | PTGS1 |
| RS15 | MOL007061 | ACHE | RS42 | MOL007125 | CHRM3 |
| RS15 | MOL007061 | ADRA1A | RS42 | MOL007125 | CHRM1 |
| RS15 | MOL007061 | CHRM2 | RS42 | MOL007125 | SCN5A |
| RS15 | MOL007061 | ADRB2 | RS42 | MOL007125 | PPARG |
| RS15 | MOL007061 | SLC6A4 | RS42 | MOL007125 | PTGS2 |
| RS15 | MOL007061 | OPRM1 | RS42 | MOL007125 | ADRA1B |
| RS15 | MOL007061 | GABRA1 | RS42 | MOL007125 | ADRB2 |
| RS15 | MOL007061 | PRSS1 | RS42 | MOL007125 | ADRA1D |
| RS15 | MOL007061 | NCOA1 | RS42 | MOL007125 | OPRM1 |
| RS16 | MOL007063 | NR3C2 | RS42 | MOL007125 | NCOA2 |
| RS16 | MOL007063 | NR3C1 | RS42 | MOL007125 | NCOA1 |
| RS17 | MOL007064 | PTGS2 | RS43 | MOL007127 | PTGS1 |
| RS17 | MOL007064 | PGR | RS43 | MOL007127 | CHRM3 |
| RS17 | MOL007064 | NR3C2 | RS43 | MOL007127 | SCN5A |
| RS17 | MOL007064 | NR3C1 | RS43 | MOL007127 | CHRM5 |
| RS17 | MOL007064 | NCOA2 | RS43 | MOL007127 | PTGS2 |
| RS17 | MOL007064 | NCOA1 | RS43 | MOL007127 | RXRA |
| RS18 | MOL007068 | PTGS2 | RS43 | MOL007127 | ADRB2 |
| RS18 | MOL007068 | RXRA | RS43 | MOL007127 | OPRM1 |
| RS18 | MOL007068 | PRSS1 | RS43 | MOL007127 | GABRA1 |
| RS18 | MOL007068 | NCOA1 | RS43 | MOL007127 | NCOA1 |
| RS19 | MOL007069 | PTGS1 | RS44 | MOL007130 | NOS2 |
| RS19 | MOL007069 | CHRM3 | RS44 | MOL007130 | PTGS1 |
| RS19 | MOL007069 | CHRM1 | RS44 | MOL007130 | ESR1 |
| RS19 | MOL007069 | SCN5A | RS44 | MOL007130 | AR |
| RS19 | MOL007069 | CHRM5 | RS44 | MOL007130 | PTGS2 |
| RS19 | MOL007069 | PTGS2 | RS44 | MOL007130 | PRSS1 |
| RS19 | MOL007069 | CHRM4 | RS45 | MOL007132 | ESR1 |
| RS19 | MOL007069 | OPRD1 | RS45 | MOL007132 | AR |
| RS19 | MOL007069 | ACHE | RS45 | MOL007132 | PPARG |
| RS19 | MOL007069 | ADRA1A | RS45 | MOL007132 | PTGS2 |
| RS19 | MOL007069 | CHRM2 | RS45 | MOL007132 | PRSS1 |
| RS19 | MOL007069 | ADRB2 | RS45 | MOL007132 | CCNA2 |
| RS19 | MOL007069 | OPRM1 | RS46 | MOL007141 | PTGS2 |
| RS19 | MOL007069 | GABRA1 | RS47 | MOL007142 | F7 |
| RS19 | MOL007069 | NCOA1 | RS47 | MOL007142 | PRSS1 |
| RS20 | MOL007070 | PTGS2 | RS48 | MOL007143 | PTGS2 |
| RS20 | MOL007070 | ACHE | RS48 | MOL007143 | RXRA |
| RS20 | MOL007070 | PRSS1 | RS48 | MOL007143 | ACHE |
| RS20 | MOL007070 | NCOA1 | RS48 | MOL007143 | PGR |
| RS21 | MOL007071 | PTGS2 | RS48 | MOL007143 | NR3C1 |
| RS21 | MOL007071 | PRSS1 | RS48 | MOL007143 | NCOA2 |
| RS21 | MOL007071 | NCOA1 | RS48 | MOL007143 | NCOA1 |
| RS22 | MOL007077 | PTGS2 | RS49 | MOL007145 | PTGS1 |
| RS23 | MOL007079 | CHRM1 | RS49 | MOL007145 | CHRM3 |
| RS23 | MOL007079 | PTGS2 | RS49 | MOL007145 | CHRM1 |
| RS23 | MOL007079 | OPRD1 | RS49 | MOL007145 | DRD5 |
| RS23 | MOL007079 | ACHE | RS49 | MOL007145 | SCN5A |
| RS23 | MOL007079 | ADRB2 | RS49 | MOL007145 | CHRM5 |
| RS23 | MOL007079 | OPRM1 | RS49 | MOL007145 | PTGS2 |
| RS23 | MOL007079 | PRSS1 | RS49 | MOL007145 | ADRA2A |
| RS23 | MOL007079 | NCOA1 | RS49 | MOL007145 | HTR3A |
| RS24 | MOL007081 | PTGS2 | RS49 | MOL007145 | CHRM4 |
| RS24 | MOL007081 | PGR | RS49 | MOL007145 | OPRD1 |
| RS24 | MOL007081 | OPRM1 | RS49 | MOL007145 | ACHE |
| RS24 | MOL007081 | NR3C1 | RS49 | MOL007145 | SLC6A2 |
| RS24 | MOL007081 | NCOA1 | RS49 | MOL007145 | ADRA1A |
| RS25 | MOL007082 | PTGS1 | RS49 | MOL007145 | CHRM2 |
| RS25 | MOL007082 | KCNH2 | RS49 | MOL007145 | ADRA2B |
| RS25 | MOL007082 | SCN5A | RS49 | MOL007145 | ADRA1B |
| RS25 | MOL007082 | PTGS2 | RS49 | MOL007145 | SLC6A3 |
| RS25 | MOL007082 | RXRA | RS49 | MOL007145 | ADRB2 |
| RS25 | MOL007082 | NCOA1 | RS49 | MOL007145 | CHRNA2 |
| RS26 | MOL007085 | PTGS1 | RS49 | MOL007145 | SLC6A4 |
| RS26 | MOL007085 | ESR1 | RS49 | MOL007145 | DRD2 |
| RS26 | MOL007085 | AR | RS49 | MOL007145 | OPRM1 |
| RS26 | MOL007085 | CHRM5 | RS49 | MOL007145 | GABRA1 |
| RS26 | MOL007085 | PTGS2 | RS49 | MOL007145 | GABRG3 |
| RS26 | MOL007085 | HTR3A | RS49 | MOL007145 | GABRE |
| RS26 | MOL007085 | ESR2 | RS50 | MOL007150 | PTGS2 |
| RS27 | MOL007088 | PTGS1 | RS50 | MOL007150 | ACHE |
| RS27 | MOL007088 | CHRM3 | RS50 | MOL007150 | PRSS1 |
| RS27 | MOL007088 | CHRM1 | RS50 | MOL007150 | NCOA1 |
| RS27 | MOL007088 | SCN5A | RS51 | MOL007151 | PTGS2 |
| RS27 | MOL007088 | CHRM5 | RS51 | MOL007151 | ACHE |
| RS27 | MOL007088 | PTGS2 | RS51 | MOL007151 | NCOA1 |
| RS27 | MOL007088 | CHRM4 | RS52 | MOL007152 | PTGS2 |
| RS27 | MOL007088 | OPRD1 | RS52 | MOL007152 | ACHE |
| RS27 | MOL007088 | ADRA1A | RS52 | MOL007152 | NCOA1 |
| RS27 | MOL007088 | CHRM2 | RS53 | MOL007154 | CHRM3 |
| RS27 | MOL007088 | ADRA1B | RS53 | MOL007154 | CHRM1 |
| RS27 | MOL007088 | ADRB2 | RS53 | MOL007154 | SCN5A |
| RS27 | MOL007088 | ADRA1D | RS53 | MOL007154 | CHRM5 |
| RS27 | MOL007088 | OPRM1 | RS53 | MOL007154 | PTGS2 |
| RS27 | MOL007088 | NCOA2 | RS53 | MOL007154 | CHRM4 |
| RS27 | MOL007088 | NCOA1 | RS53 | MOL007154 | OPRD1 |
| RS27 | MOL007088 | PGR | RS53 | MOL007154 | ACHE |
| RS27 | MOL007088 | GABRA1 | RS53 | MOL007154 | ADRA1A |
| RS27 | MOL007088 | RELA | RS53 | MOL007154 | CHRM2 |
| RS27 | MOL007088 | STAT3 | RS53 | MOL007154 | ADRB2 |
| RS27 | MOL007088 | CCND1 | RS53 | MOL007154 | OPRM1 |
| RS27 | MOL007088 | BCL2L1 | RS53 | MOL007154 | NCOA1 |
| RS27 | MOL007088 | TNFSF15 | RS53 | MOL007154 | RXRA |
| RS27 | MOL007088 | APP | RS53 | MOL007154 | RELA |
| RS27 | MOL007088 | EDN3 | RS53 | MOL007154 | BCL2 |
| RS27 | MOL007088 | BIRC5 | RS53 | MOL007154 | FOS |
| RS28 | MOL007093 | NOS2 | RS53 | MOL007154 | CDKN1A |
| RS28 | MOL007093 | PTGS1 | RS53 | MOL007154 | MMP9 |
| RS28 | MOL007093 | KCNH2 | RS53 | MOL007154 | JUN |
| RS28 | MOL007093 | CHRM1 | RS53 | MOL007154 | AHSA1 |
| RS28 | MOL007093 | ESR1 | RS53 | MOL007154 | CASP3 |
| RS28 | MOL007093 | AR | RS53 | MOL007154 | TP63 |
| RS28 | MOL007093 | SCN5A | RS53 | MOL007154 | NFKBIA |
| RS28 | MOL007093 | PPARG | RS53 | MOL007154 | FASN |
| RS28 | MOL007093 | PTGS2 | RS53 | MOL007154 | EDNRA |
| RS28 | MOL007093 | RXRA | RS53 | MOL007154 | EDN3 |
| RS28 | MOL007093 | ACHE | RS53 | MOL007154 | CYP3A4 |
| RS28 | MOL007093 | ADRA1B | RS53 | MOL007154 | CYP1A2 |
| RS28 | MOL007093 | ADRB2 | RS53 | MOL007154 | MYC |
| RS28 | MOL007093 | ESR2 | RS53 | MOL007154 | CYP1A1 |
| RS28 | MOL007093 | GSK3B | RS53 | MOL007154 | NR1I2 |
| RS28 | MOL007093 | CHEK1 | RS53 | MOL007154 | NPM1 |
| RS28 | MOL007093 | PRSS1 | RS53 | MOL007154 | ECE1 |
| RS28 | MOL007093 | CCNA2 | RS53 | MOL007154 | PARP4 |
| RS28 | MOL007093 | NCOA2 | RS53 | MOL007154 | CALCR |
| RS28 | MOL007093 | NCOA1 | RS53 | MOL007154 | ITGB3 |
| RS29 | MOL007094 | PTGS1 | RS54 | MOL007155 | CHRM1 |
| RS29 | MOL007094 | CHRM3 | RS54 | MOL007155 | SCN5A |
| RS29 | MOL007094 | CHRM1 | RS54 | MOL007155 | PTGS2 |
| RS29 | MOL007094 | ESR1 | RS54 | MOL007155 | OPRD1 |
| RS29 | MOL007094 | SCN5A | RS54 | MOL007155 | ACHE |
| RS29 | MOL007094 | CHRM5 | RS54 | MOL007155 | ADRA1A |
| RS29 | MOL007094 | PTGS2 | RS54 | MOL007155 | ADRB2 |
| RS29 | MOL007094 | CHRM4 | RS54 | MOL007155 | OPRM1 |
| RS29 | MOL007094 | RXRA | RS54 | MOL007155 | PRSS1 |
| RS29 | MOL007094 | ACHE | RS54 | MOL007155 | NCOA1 |
| RS29 | MOL007094 | ADRA1A | RS55 | MOL007156 | PTGS1 |
| RS29 | MOL007094 | CHRM2 | RS55 | MOL007156 | ESR1 |
| RS29 | MOL007094 | ADRA1B | RS55 | MOL007156 | AR |
| RS29 | MOL007094 | ADRB2 | RS55 | MOL007156 | SCN5A |
| RS29 | MOL007094 | ADRA1D | RS55 | MOL007156 | PPARG |
| RS29 | MOL007094 | CHRNA2 | RS55 | MOL007156 | PTGS2 |
| RS29 | MOL007094 | SLC6A4 | RS55 | MOL007156 | NCOA2 |
| RS29 | MOL007094 | OPRM1 | RS55 | MOL007156 | NCOA1 |
