# Supplementary material for: A System Bioinformatics Approach Predicts the Molecular Mechanism Underlying the Course of Action of Radix Salviae Reverses GBM Effects
Source: Evid Based Complement Alternat Med. 2021 Dec 16;2021:1218969. doi: 10.1155/2021/1218969 (PMC8825271; doi:10.1155/2021/1218969)
Supplement: Supplementary Materials — Supplementary Table 1: potential active components of Radix Salviae. Supplementary Table 2: molecular information and targets corresponding to the coding ID. [file 1218969.f1.zip › 1218969.f1/Supplementary Table 1..doc]

| MOLID | Molecule | OB(%) | DL(%) |
| --- | --- | --- | --- |
| MOL001601 | 1,2,5,6-tetrahydrotanshinone | 38.75 | 0.36 |
| MOL001659 | Poriferasterol | 43.83 | 0.76 |
| MOL001771 | poriferast-5-en-3beta-ol | 36.91 | 0.75 |
| MOL001942 | isoimperatorin | 45.46 | 0.23 |
| MOL002222 | sugiol | 36.11 | 0.28 |
| MOL002651 | Dehydrotanshinone II A | 43.76 | 0.4 |
| MOL002776 | Baicalin | 40.12 | 0.75 |
| MOL000569 | digallate | 61.85 | 0.26 |
| MOL000006 | luteolin | 36.16 | 0.25 |
| MOL006824 | α-amyrin | 39.51 | 0.76 |
| MOL007036 | 5,6-dihydroxy-7-isopropyl-1,1-dimethyl-2,3-dihydrophenanthren-4-one | 33.77 | 0.29 |
| MOL007041 | 2-isopropyl-8-methylphenanthrene-3,4-dione | 40.86 | 0.23 |
| MOL007045 | 3α-hydroxytanshinoneⅡa | 44.93 | 0.44 |
| MOL007048 | (E)-3-[2-(3,4-dihydroxyphenyl)-7-hydroxy-benzofuran-4-yl]acrylic acid | 48.24 | 0.31 |
| MOL007049 | 4-methylenemiltirone | 34.35 | 0.23 |
| MOL007050 | 2-(4-hydroxy-3-methoxyphenyl)-5-(3-hydroxypropyl)-7-methoxy-3-benzofurancarboxaldehyde | 62.78 | 0.4 |
| MOL007051 | 6-o-syringyl-8-o-acetyl shanzhiside methyl ester | 46.69 | 0.71 |
| MOL007058 | formyltanshinone | 73.44 | 0.42 |
| MOL007059 | 3-beta-Hydroxymethyllenetanshiquinone | 32.16 | 0.41 |
| MOL007061 | Methylenetanshinquinone | 37.07 | 0.36 |
| MOL007063 | przewalskin a | 37.11 | 0.65 |
| MOL007064 | przewalskin b | 110.32 | 0.44 |
| MOL007068 | Przewaquinone B | 62.24 | 0.41 |
| MOL007069 | przewaquinone c | 55.74 | 0.4 |
| MOL007070 | (6S,7R)-6,7-dihydroxy-1,6-dimethyl-8,9-dihydro-7H-naphtho[8,7-g]benzofuran-10,11-dione | 41.31 | 0.45 |
| MOL007071 | przewaquinone f | 40.31 | 0.46 |
| MOL007077 | sclareol | 43.67 | 0.21 |
| MOL007079 | tanshinaldehyde | 52.47 | 0.45 |
| MOL007081 | Danshenol B | 57.95 | 0.56 |
| MOL007082 | Danshenol A | 56.97 | 0.52 |
| MOL007085 | Salvilenone | 30.38 | 0.38 |
| MOL007088 | cryptotanshinone | 52.34 | 0.4 |
| MOL007093 | dan-shexinkum d | 38.88 | 0.55 |
| MOL007094 | danshenspiroketallactone | 50.43 | 0.31 |
| MOL007098 | deoxyneocryptotanshinone | 49.4 | 0.29 |
| MOL007100 | dihydrotanshinlactone | 38.68 | 0.32 |
| MOL007101 | dihydrotanshinoneⅠ | 45.04 | 0.36 |
| MOL007105 | epidanshenspiroketallactone | 68.27 | 0.31 |
| MOL007107 | C09092 | 36.07 | 0.25 |
| MOL007108 | isocryptotanshi-none | 54.98 | 0.39 |
| MOL007111 | Isotanshinone II | 49.92 | 0.4 |
| MOL007115 | manool | 45.04 | 0.2 |
| MOL007118 | microstegiol | 39.61 | 0.28 |
| MOL007119 | miltionone Ⅰ | 49.68 | 0.32 |
| MOL007120 | miltionone Ⅱ | 71.03 | 0.44 |
| MOL007121 | miltipolone | 36.56 | 0.37 |
| MOL007122 | Miltirone | 38.76 | 0.25 |
| MOL007123 | miltirone Ⅱ | 44.95 | 0.24 |
| MOL007124 | neocryptotanshinone ii | 39.46 | 0.23 |
| MOL007125 | neocryptotanshinone | 52.49 | 0.32 |
| MOL007127 | 1-methyl-8,9-dihydro-7H-naphtho[5,6-g]benzofuran-6,10,11-trione | 34.72 | 0.37 |
| MOL007130 | prolithospermic acid | 64.37 | 0.31 |
| MOL007132 | (2R)-3-(3,4-dihydroxyphenyl)-2-[(Z)-3-(3,4-dihydroxyphenyl)acryloyl]oxy-propionic acid | 109.38 | 0.35 |
| MOL007140 | (Z)-3-[2-[(E)-2-(3,4-dihydroxyphenyl)vinyl]-3,4-dihydroxy-phenyl]acrylic acid | 88.54 | 0.26 |
| MOL007141 | salvianolic acid g | 45.56 | 0.61 |
| MOL007142 | salvianolic acid j | 43.38 | 0.72 |
| MOL007143 | salvilenone Ⅰ | 32.43 | 0.23 |
| MOL007145 | salviolone | 31.72 | 0.24 |
| MOL007149 | NSC 122421 | 34.49 | 0.28 |
| MOL007150 | (6S)-6-hydroxy-1-methyl-6-methylol-8,9-dihydro-7H-naphtho[8,7-g]benzofuran-10,11-quinone | 75.39 | 0.46 |
| MOL007151 | Tanshindiol B | 42.67 | 0.45 |
| MOL007152 | Przewaquinone E | 42.85 | 0.45 |
| MOL007154 | tanshinone iia | 49.89 | 0.4 |
| MOL007155 | (6S)-6-(hydroxymethyl)-1,6-dimethyl-8,9-dihydro-7H-naphtho[8,7-g]benzofuran-10,11-dione | 65.26 | 0.45 |
| MOL007156 | tanshinone Ⅵ | 45.64 | 0.3 |
